# An intense ^18^F-FDG pulmonary microfocus on PET without detectable abnormality on CT: A manifestation of an iatrogenic FDG pulmonary embolus

**DOI:** 10.2349/biij.6.4.e37

**Published:** 2010-10-01

**Authors:** AS Fathinul Fikri, WFE Lau

**Affiliations:** 1 Nuclear Imaging Centre, Faculty of Medicine, University Putra Malaysia; 2 Department of Radiology, The University of Melbourne and Centre for Molecular Imaging, The Peter MacCallum Cancer Centre, Australia

**Keywords:** 18-FDG pulmonary embolus, iatrogenic, radiolabelled-RBC

## Abstract

An incidental finding of an intense focus of ^18^F-Fluorodeoxyglucose (FDG) pulmonary uptake on positron emission tomography (PET) without detectable lesions on computed tomography (CT) is highly suggestive of FDG microembolus. Its microscopic nature means it is undetectable on CT. It is an artefact attributable to ^18^F-FDG-tracer contamination at the injection site. This paper reports a case of a 61 year-old lady with a past history of breast carcinoma, in whom follow-up PET/CT images demonstrated an incidental intense FDG pulmonary abnormality. A follow-up PET/CT seven months later demonstrated complete resolution of the abnormality.

## INTRODUCTION

Positron Emission Tomography/Computed Tomography (PET/CT) fusion imaging is a novel multi-modality technology that allows the correlation of findings from two concurrent imaging modalities in a comprehensive examination. As altered glucose metabolism is characteristic for many malignancies, FDG-PET is mostly used in oncology for staging and therapy monitoring. It can also be seen as a physiologic activity in the bowel or muscles, in benign disease such as inflammation or post-traumatic reactive changes, or simply represent artefactual uptake from inaccurate CT attenuation correction in dense objects, often mistaken for cancer [1–3].

The experienced PET/CT reader mostly manages to differentiate malignant from non-malignant FDG uptake based on accurate interpretation of the PET data by correlating with the CT anatomic information [1–3]. This paper documents an example of a false positive PET/CT interpretation of an artefact, which may at times be erroneously diagnosed as a pathological entity.

## CASE REPORT 1

**Figure 1:**
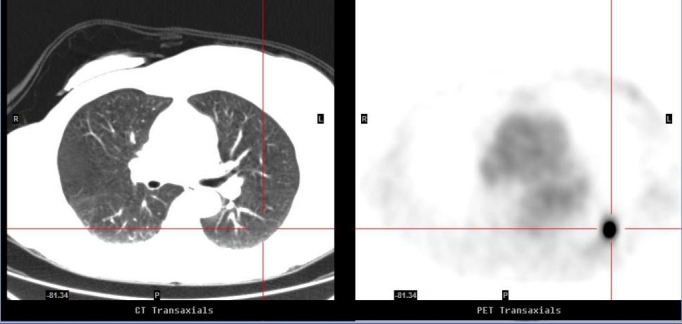
Correlative axial images of CT (left) and PET (right) (February 2007): The PET image displayed an intense ^18^F-FDG focus in the apical segment of the left lower lobe which is imperceptible on the corresponding CT image. It has maximal SUV of 17.9 and an estimated maximal diameter of 2.5 cm.

**Figure 2:**
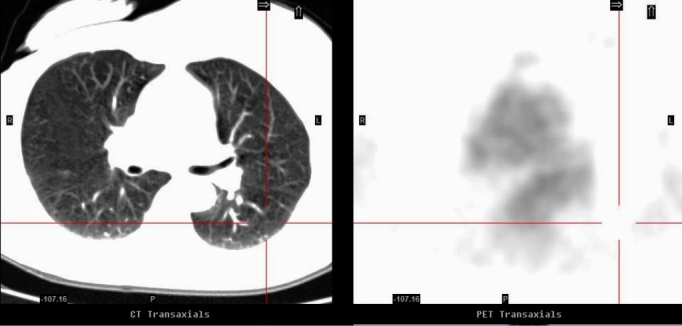
Correlative axial images of CT (left) and PET (right) (September 2007): The previously documented focal FDG uptake in the left lung has resolved, likely to represent FDG microembolus rather than metastatic disease.

This case report looks at a 61 year-old lady who has a history of nodal positive right breast cancer treated by surgery and chemotherapy in 1996. She was referred for a PET/CT in February 2007 with the query of locoregional recurrence because of recurrent chest wall pain. PET/CT study revealed an intense ^18^F-FDG focus in the apical segment of the left lower lobe on PET without any perceptible corresponding CT lesion (Figure 1). By virtue of the lesion-imperceptibility on the correlative CT, a provisional diagnosis of an iatrogenic FDG microembolus was made and the patient was managed conservatively. A follow-up PET/CT done seven months later demonstrated complete resolution of the PET abnormality and confirmed its benign aetiology (Figure 2).

## DISCUSSION

In this case, the intense ^18^F-FDG focus in the left lower lobe is highly suggestive of thromboembolus phenomenon attributed to a tagged platelet or mixed nature rather than red blood cells [3, 5]. This detected FDG activity was no longer apparent on the follow-up PET/CT done seven months later. Of late, there have been a number of similar reported cases and all the cases exhibited similar features on the PET/CT correlative images [1–3].

An intense ^18^F-FDG-PET focus without a detectable CT abnormality may rarely be a result of an early imperceptible structural lesion [1–4]. Complete resolution of such an intense FDG focus in all the known reported instances is, therefore, essential to establish the final diagnosis.

In principle, an intense FDG focus is not entirely specific for malignancy. In general, CT is more sensitive than FDG-PET in the detection of pulmonary lesions, given its superior spatial resolution [4]. Therefore the absence of structural lesions on CT alerts to the possibility that the focal FDG-PET intensity is not a genuine abnormality.

This is in line with the fact that more than one-third of scintigraphic equivocal lesions were correctly triaged by analysis of the morphological and anatomical details on the correlative CT study [4].

Incidence of microembolus formation involving radiotracer injection has also been reported in patients with abnormal peripheral venous system and difficult phlebotomy, following the usage of an old intravenous cannula, or the presence of a hypercoagulability state in which the FDG is mixed with blood products prior to injection [5]. The phenomenon may be reduced by using a new intravenous cannula, avoiding the withdrawal of blood prior to FDG injection and adopting a good intravenous technique.

## CONCLUSION

An intense ^18^F-FDG-PET pulmonary abnormality without detectable lesions on the correlative CT study is highly suggestive of artefacts caused by FDG microembolus. This report emphasises the importance of becoming familiar with the potential pitfalls of false-positive interpretations of PET.
